# Alignment-free clustering of large data sets of unannotated protein conserved regions using minhashing

**DOI:** 10.1186/s12859-018-2080-y

**Published:** 2018-03-05

**Authors:** Armen Abnousi, Shira L. Broschat, Ananth Kalyanaraman

**Affiliations:** 1 School of EECS, Washington State University, 355 NE Spokane St, Pullman, 99164 USA; 2 Paul G. Allen School for Global Animal Health, Washington State University, Pullman, 99164 USA; 3 Department of Veterinary Microbiology and Pathology, Washington State University, Pullman, 99164 USA

**Keywords:** Protein conserved region, Clustering, Protein domain families

## Abstract

**Background:**

Clustering of protein sequences is of key importance in predicting the structure and function of newly sequenced proteins and is also of use for their annotation. With the advent of multiple high-throughput sequencing technologies, new protein sequences are becoming available at an extraordinary rate. The rapid growth rate has impeded deployment of existing protein clustering/annotation tools which depend largely on pairwise sequence alignment.

**Results:**

In this paper, we propose an alignment-free clustering approach, coreClust, for annotating protein sequences using detected conserved regions. The proposed algorithm uses Min-Wise Independent Hashing for identifying similar conserved regions. Min-Wise Independent Hashing works by generating a (w,c)-sketch for each document and comparing these sketches. Our algorithm fits well within the MapReduce framework, permitting scalability. We show that coreClust generates results comparable to existing known methods. In particular, we show that the clusters generated by our algorithm capture the subfamilies of the Pfam domain families for which the sequences in a cluster have a similar domain architecture. We show that for a data set of 90,000 sequences (about 250,000 domain regions), the clusters generated by our algorithm give a 75% average weighted *F*1 score, our accuracy metric, when compared to the clusters generated by a semi-exhaustive pairwise alignment algorithm.

**Conclusions:**

The new clustering algorithm can be used to generate meaningful clusters of conserved regions. It is a scalable method that when paired with our prior work, NADDA for detecting conserved regions, provides a complete end-to-end pipeline for annotating protein sequences.

**Electronic supplementary material:**

The online version of this article (10.1186/s12859-018-2080-y) contains supplementary material, which is available to authorized users.

## Background

Proteins play a fundamental role in living organisms, from their various responsibilities in metabolic pathways to transporting molecules within the cell. Understanding the mechanisms of a cell requires a clear insight into the structure and roles of the proteins in the cell. However, new approaches to sequencing have resulted in a growing number of protein sequences being generated and stored in databases; the rate of the increase has outpaced our ability to manually examine the generated proteins. As an example of such growth, the UniProt knowledgebase for proteins [1] contains over 90 million protein sequences, but of this number only 550,000 have been curated by experts^1^ (using both experimental and predicted data). This rapid growth rate has in turn created a growing need to develop automated methods.

Proteins are comprised of evolutionary blocks known as domains [2]. Clustering proteins based on these domains is a key to predicting protein function and structure. In fact, functional annotation of the *Caenorhabditis elegans* genome was one of the primary drivers leading to the design of the well-known Pfam protein family database [3]. Two proteins that have a common domain should be assigned to the same cluster. Because each sequence can contain multiple domains, it can also belong to different protein clusters.

In previous work we introduced NADDA [4], an alignment-free method for detection of protein conserved regions. Given a set of protein sequences, NADDA detects subsequences that are likely to belong to a conserved region, hence fragmenting the proteins into shorter conserved regions. However, NADDA does not annotate these regions, but rather it merely reports them as conserved.

In this paper, we present coreClust^2^, a clustering method based on detected conserved regions. Detection of such regions point to domains, which can subsequently be used for functionally annotating and grouping protein sequences. coreClust is based on a technique called MinHash [5] which is a locality-sensitive hashing approach for identifying similar elements in a set [6, 7]. Because it is mainly dependent on hashing, our method fits well within the MapReduce[8] parallel processing platform, permitting scalability.

After a brief discussion of previous work done in the field, in the next section we describe our approach to clustering conserved regions and generation of protein clusters. Then in the Results section, we present our cluster evaluation and runtime analysis as well as a brief case-study utilizing our approach. Finally a discussion of the observations, limitations of the method, and conclusions are presented.

### Related work

As mentioned earlier, various clustering approaches for proteins have been proposed over the years. However most of these methods depend on pairwise sequence similarity between proteins in a set. Similarity scores traditionally can be computed using dynamic programming algorithms such as Needleman-Wunsch [9] for global similarity and Smith-Waterman [10] for local similarity. These algorithms have quadratic time complexity in the length of the sequences, imposing severe limitations on the size of the sets to which they can be applied. As an alternative to these methods, other similarity methods such as Basic Local Alignment Search Tool (BLAST) and its variants [11, 12] have been proposed. However, BLAST is a heuristic approach invented for efficient database search (i.e. searching a small number of queries against a large database). For our use-case, we need an efficient method that can effectively perform all-against-all sequence comparisons and use the results to group protein sequences by their shared domains. Such an operation can be highly expensive, and BLAST-based tools have been shown to be ineffective under such settings [13]. Instead, recent focus has shifted towards alignment-free methods [14].

Protein clustering methods can be categorized into five groups: motif-based, full-sequence analysis, phylogenetic classification, structure-dependent, and aggregated methods [15]. The methods in the motif-based category, being dependent on domains and motifs, allow generation of overlapping clusters of proteins. This leads to clusters with high-resolution, and hence these methods are more accurate. Our method together with our previous work (NADDA) falls under this latter category.

Arguably, most of the methods in the motif-based category perform more as a classification method rather than as a clustering method in the sense that they depend on known families of proteins. They construct various representatives for the known families, such as regular expressions or hidden Markov models, and then given one (or a set of) query protein they compare this sequence with the constructed models and place it in the family that gives the best match. Examples of these methods are Pfam [3, 16], SMART [17, 18], PROSITE [19], PRINTS [20] and TIGRFAMs [21].

On the other hand, there are a few methods that try to automatically generate conserved regions or an estimate of these regions and perform the clustering based on them. These methods are more similar to our proposed approach. Examples of these methods are EVEREST [22], ADDA [23], DOMO [24], and pClust [25] and its derivatives. However all of these methods depend on pairwise sequence alignment, either on the entire set of input sequences or on some subsets of the input that are selected using various filtering approaches.

Everest performs an all-vs-all BLAST of the complete data set (using the data set itself as the BLAST database) followed by Smith-Waterman sequence alignment for the selected sequences from the BLAST results to construct a set of putative domain regions. It then performs clustering of the putative domain regions and HMM profiles are built for the high-scoring clusters. These profiles are used to look for similar regions in the original data set, the result of which replaces the initial putative domains. This operation is repeated iteratively, each time refining the HMM profiles and the resulting clusters.

ADDA uses an optimization approach to detect the borders of the domain regions. ADDA first generates a sequence space graph by performing an all-against-all BLAST on the entire data set of sequences. The nodes on the sequence space graph represent the sequences, and edges are alignments between sequences based on the BLAST results. From this graph, trees of putative domains (a set of nested putative domains) are constructed by repetitively splitting a “residue correlation matrix” into two submatrices. After generation of the tree of the putative domains for each sequence, an optimization target is used to select the optimal domains for all sequences simultaneously (i.e., with regard to each other). Based on detected boundaries, the sequence space graph is converted into a domain graph. After some computation on the domain graph, such as computing the minimum spanning tree, each component of the tree is output as one protein family.

DOMO and pClust depend on preliminary computation to filter out sequences that do not appear to be similar to each other to reduce the computation required for multiple sequence alignment. In DOMO, the authors use a composition similarity search (where two sequences are considered similar if the amino acid and dipeptide composition distance between them is below a pre-defined threshold), followed by construction of a suffix tree to detect groups of sequences that have higher local similarities. Then using pairwise similarities they choose the domain boundaries [26]. Finally these domains are clustered together based on shared similarities.

Although similar to our approach pClust uses min-hashing in its operation, first using a Generalized Suffix Tree to find pairs of sequences that have a significantly long maximal match, then performing sequence alignment on these pairs to decide whether they should really be considered similar. This process results in construction of a sequence similarity graph. For each connected component of this similarity graph, it constructs a bipartite graph, where on the left side the nodes represent sequences and on the right side the nodes represent *w*-length substrings present in at least two different sequences on the left side. An edge connecting a node from left to right shows the presence of the substring on the right in the sequence node on the left. After this operation pClust performs dense subgraph detection using a min-hash locality-sensitive hashing algorithm [5, 27].

As can be seen, all of the methods described above depend on pairwise sequence alignment or a variant of BLAST on either the complete data set or on subsets selected by applying filters such as generalized suffix trees. coreClust avoids the need for any sequence alignment operation by first constructing a similarity graph using min-hashing and then applying a clustering method on the generated graph to find the final clusters.

## Methods

The problem addressed by our method can be defined as follows: The input is a set of *n* protein sequences such that each sequence is marked with a set of one or more conserved regions; for the purpose of computation, a *conserved region* within a sequence *s* corresponds to a substring of *s*. Given this input, the problem of clustering is one of grouping the protein sequences into (possibly overlapping) “clusters” such that all sequences that contain the same conserved region are mapped to one cluster. Note that the containment is based on similarity (as opposed to identity) of the conserved regions—i.e., two copies of the same conserved region in two different sequences are expected to be highly similar but not necessarily identical. While this goal can be achieved by performing all-against-all protein sequence comparisons via alignments, we want to achieve the goal without requiring such all-against-all comparisons or alignments.

In previous work [4] we developed an alignment-free method for detection of conserved regions in protein sequences. Here we focus on using detected conserved regions to generate clusters that satisfy the requirement stated above. In order to generate clusters from the conserved regions we propose an iterative two-step clustering algorithm. In the first step of each iteration, we use min-wise independent hashing (min-hashing) [5] to generate a similarity graph, and in the second step we use the Louvain method for community detection [28] to generate clusters from the generated similarity graph. In what follows, we discuss each of these steps and the iteration in detail. The pseudo-code for the overall approach is shown in Algorithm 1.

### Min-wise independent hashing

The intuition behind min-wise independent hashing is that rather than comparing the entirety of two documents to decide whether they are similar, we first pick a sample from the two documents and compare them.

In [5, 29], the authors show that there exists a sampling function *L* such that for two documents *D*_1_ and *D*_2_, the Jaccard similarity between *L*(*D*_1_) and *L*(*D*_2_) is an unbiased estimate of the Jaccard similarity between *D*_1_ and *D*_2_. The sampling function they propose depends on a random permutation of the terms in the document. In [30], the authors introduce a min-wise independent family of permutations and show that it suffices to select a permutation from this family. They also show that a linear permutation of form *ax*+*b* mod *p*, although not min-wise independent, works well in practice. In [27], the authors use this family of linear permutations for discovering dense subgraphs.

Min-Wise Independent Hashing works by generating a *(w,c)-sketch* for each document and comparing these sketches [27, 29]. Two documents are considered to be similar if their *(w,c)-sketch*es are equal. To generate a *(w,c)-sketch* we compute all possible *w-shingle*s for a document by hashing contiguous sequences of length *w* of the words in the document using a min-wise independent hashing function (or its substitute, e.g., a linear permutation as explained above) and concatenating the *c* minimum terms from the results. Documents might exist that have dissimilar sketches and thus are not paired together while in reality they are similar. To avoid such incidences, we can repeat the same operation multiple times using different permutation functions to compute the sketches. On the other hand, there might be some documents that are paired as similar due to the equality of their *(w,c)-sketch*es while in reality they are not similar. To filter out these false positive instances, we can repeat the entire operation and compute sketches of the sketches using hash functions that differ from those used in the first iteration. Then if the second-level sketches of two documents are equal, we accept the decision that the two documents are similar; otherwise we reject the decision. This operation can be repeated iteratively multiple times, but it has been shown that in practice two iterations suffice [27].

### Similarity graph construction via min-wise independent hashing

We use Min-Wise Independent Hashing using linear transformations of form *ax*+*b* mod *p* to find conserved regions in the input data set that are similar to each other and construct a similarity graph based on this. The process of similarity graph construction for conserved regions differs from the one explained above in two ways. First, for conserved regions, rather than applying the linear transformation on the contiguous sequences of *w* words from the documents, we apply the hash functions on the subsequences of length *k* of each protein sequence, known as *k*-mers of the protein sequences (line 1 in Algorithm 2). Second, rather than computing the second-level sketches from the first-level sketches, we generate an initial similarity graph from the first-level sketches (where nodes are conserved regions and an edge between two nodes represents the potential similarity between their corresponding conserved regions) and then apply the same min-hashing algorithm on this graph rather than on the first-level sketches. In other words, to generate the second-level sketches of conserved region *s*, rather than applying the hash functions on the first-level sketches of *s*, we apply the hash functions on the set of neighbors of *s*, i.e., on the names of the conserved regions that were deemed potentially similar to *s* (line 16 in Algorithm 2) based on the first-level sketches. If two nodes in the initial graph share a majority of their neighbors, they will likely have an equal second-level sketch. We construct a new similarity graph based on the results of the second-level sketches. The graph constructed using the second-level sketches can be interpreted in the same way as the initial graph, i.e., nodes represent conserved regions, and there exists an edge between two nodes if and only if the two conserved regions corresponding to these nodes are similar based on our method.

**Fig. 1:**
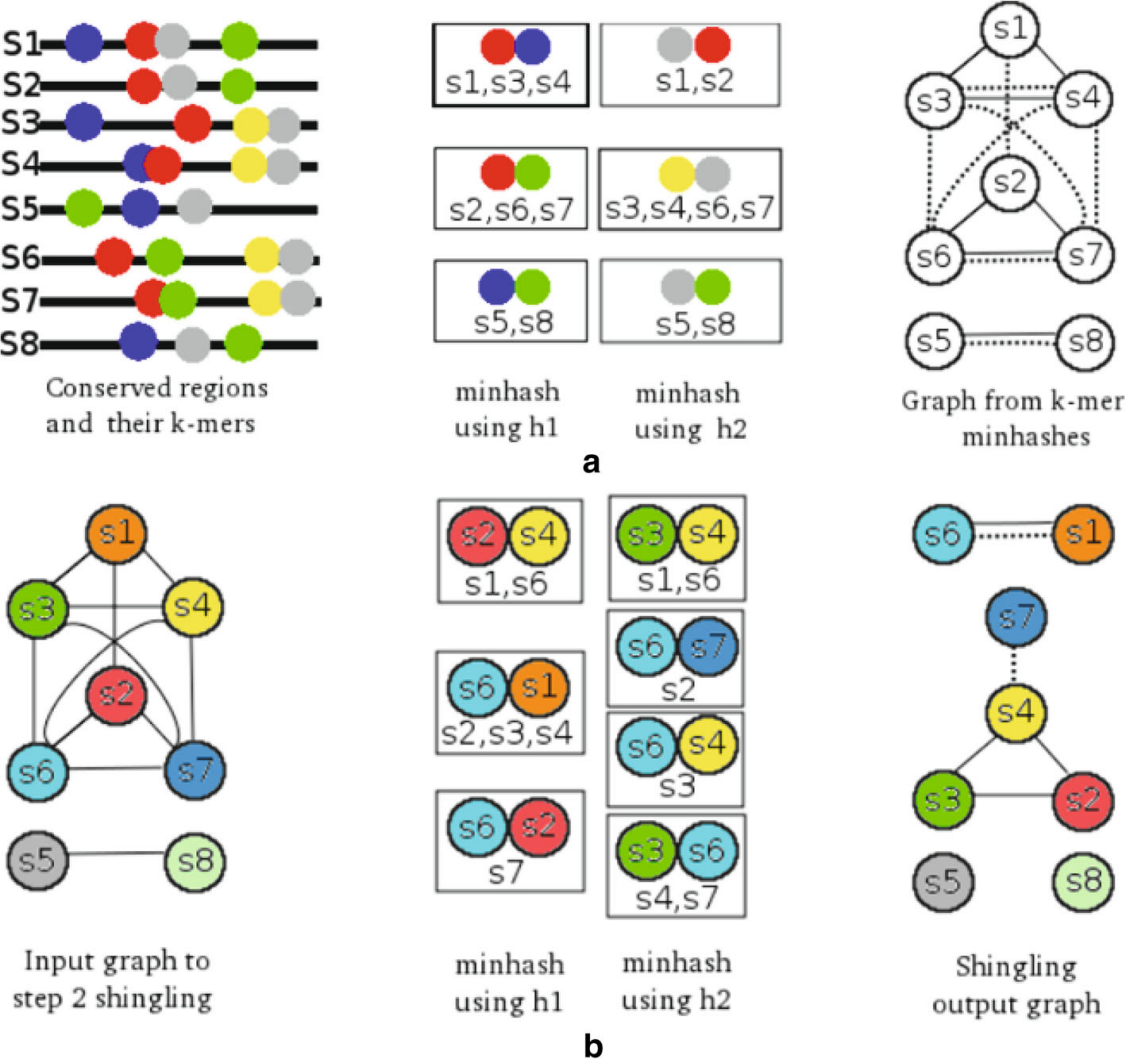
Construction of the similarity graph. In the first step, *h*1(*red*)<*h*1(*blue*)<*h*1(*green*)<*h*1(*gray*)<*h*1(*yellow*) and *h*2(*yellow*)<*h*2(*gray*)<*h*2(*red*)<*h*2(*green*)<*h*2(*blue*). In the second step, *h*1(6)<*h*1(1)<*h*1(2)<*h*1(4)<*h*1(3)<*h*1(7) and *h*2(3)<*h*2(4)<*h*2(1)<*h*2(6)<*h*2(7)<*h*2(2). In the graph output at each step, solid lines represent edges generated by *h*1 and dashed lines represent edges generated by *h*2. **a** First step shingling, based on conserved region k-mears. **b** Second step shingling

Figure 1 demonstrates the graph construction process for 8 conserved regions using a sketch of size two and two hash functions. In Fig. 1a, the conserved regions are shown using lines and a subset of their *k*-mers using circles of different colors. We have assumed that these *k*-mers are the ones that give the minimum sketch for each conserved region using the two hash functions *h*1 and *h*2. For example, for the conserved region *s*2, applying *h*1 generates the *<red, green>* pair as its first minhash or *(k,2)-sketch* because we have assumed the ordering: *h*1(*red*)<*h*1(*blue*)<*h*1(*green*)<*h*1(*gray*)<*h*1(*yellow*). Similarly, the pair *<gray, red>* will be the minhash for *s*2 using the second hash function *h*2. Because *s*2 and *s*6 have the common sketch *<red, green>* from applying *h*1 on their *k*-mers, there is an edge between the two nodes corresponding to the two conserved regions. On the other hand since *s*2 shares its sketch generated by *h*2 with *s*1, there is another edge connecting the two nodes in the resulting initial graph (demonstrated by a dashed line). For generation of the second-level sketches (Fig. 1b) we ignore the information regarding the hash function that resulted in the generation of an edge (i.e., disregard the difference between the dashed and solid lines in the output graph of Fig. 1a and allow at most one edge between every pair of nodes) and use this consolidated graph as input, applying the hash functions on the set of neighbors of each node. For node *s*2, applying *h*1 on its set of neighbors *s*1,*s*6,*s*7 gives the sketch *< s6, s1>* because we have assumed that *h*1(6)<*h*1(1)<*h*1(7), and applying *h*2 results in the sketch *< s6, s7>*. The first sketch yields edges between the nodes corresponding to conserved regions *s*2, *s*3, and *s*4 (shown by a solid line in the output graph for the shingling), while the second sketch, *< s6, s7>*, does not result in any edges because *s*2 is the only node with this sketch from *h*2 (hence, no dashed line connected to *s*2 in the final graph). The consolidated graph generated from the second-level sketches is the similarity graph that we will use in the next step of our algorithm.

We use *c*=2 and *k*=6 in the computation of our first level sketches, i.e., for the first-level sketches we apply hash functions on subsequences of length 6 of each conserved region and concatenate two minimum values computed using each hash function for each conserved region to generate a sketch (line 11 in Algorithm 2). For the generation of second-level sketches we use *c*=2 and *k*=1, i.e., for each node we hash all its neighbors’ names using a hash function and select the two that give the minimum hash value. The concatenated names of those two neighbors gives the sketch generated for that conserved region (line 26 in Algorithm 2).





### Community detection on the similarity graph

After we have generated a similarity graph for the conserved regions, we need to cluster the graph such that there are a relatively large number of edges within each cluster compared to the number of edges between two separate clusters. This is a well-studied problem. For this purpose, we use the Louvain method for community detection on the constructed similarity graph.

The Louvain method for community detection is based on the modularity of the clustering. Modularity is defined as follows:

Given a partitioning *P* of a graph with node set *V*, where a node *i* is assigned to partition *C*(*i*), the modularity of a clustering is measured as:

$$Q = \frac{1}{2m}\sum\limits_{i \in V} e_{i\rightarrow C(i)} - \sum\limits_{C \in P}\left(\frac{a_{C}}{2m}.\frac{a_{C}}{2m}\right)$$

Where *m* is the sum of the edge weights; *C* is used to represent one partition from the partitioning *P*; *C*(*i*) represents the partition that contains node *i*; *e*_*i*→*C*(*i*)_ denotes the sum of the edge weights for the edges between the node *i* and other nodes inside the same partition as *i* (inside *C*(*i*)); and *a*_*C*_ is the sum of the degrees of the nodes in partition *C*. A partitioning is considered good if the corresponding modularity value is high.

Based on this definition for modularity, the Louvain method measures the net modularity gain by moving one node from its current partition to a neighboring partition (a partition that contains one of the neighbors of the node). The operation stops if none of the neighboring partitions gives a positive net modularity gain or after a fixed number of iterations. In our experiments we have used Grappolo [31], a multi-threaded implementation of the Louvain method.

### Iterative clustering

As described earlier for the “Min-wise independent hashing” method, using one hash function is a rather conservative approach and will likely result in detection of some but not all of the similar conserved regions. However, the best number of hash functions required for construction of a similarity graph that is a true representation of the input data set is a function of the degree of conservation among the input sequences and, hence, while using a fixed number of hash functions might work well for one set, it may be too few for another set. To overcome this problem in our method we propose starting with a small number of hash functions, *h*, (e.g., *h*=41) and continue adding to the number of hash functions gradually until a termination condition is met. In each step, we complete the clustering and compare the results with the one achieved using *h*−*d* hash functions, for a fixed *d* (e.g., *d*=40). If the comparison shows that the two clusters are, for the most part, similar to each other, we stop the iterations and the last generated set of clusters is output as the final result. Otherwise, if the similarity between the two clusters is not high enough, we increment the number of hash functions by one (or by another fixed small number). Comparison of the two sets of clusters to decide their degree of similarity can be performed by measuring the average weighted *F*1 score described below. By this arrangement, the termination condition depends on two parameters, a distance value *d* which is the difference between the number of hash functions used in generation of two clusters in each iteration and a threshold value *τ* for the *F*1 score.

### Comparison of two sets of clusters using *F*1 score

Let *X*_*i*_ and *Y*_*j*_ be two clusters of sizes |*X*_*i*_| and |*Y*_*j*_|, respectively, from the two clusterings *X* and *Y*. Then we define the *precision*(*X*_*i*_→*Y*_*j*_) and *recall*(*X*_*i*_→*Y*_*j*_) as:

$$\begin{aligned} precision(X_{i} \rightarrow Y_{j}) = \frac{|X_{i} \cap Y_{j}|}{|Y_{j}|}\\ recall(X_{i} \rightarrow Y_{j}) = \frac{|X_{i} \cap Y_{j}|}{|X_{i}|} \end{aligned} $$

Then for cluster *X*_*i*_ from clustering *X* we can measure its resemblance to a best counterpart in *Y* with regard to *precision* and *recall* by:

$$\begin{array}{@{}rcl@{}} precision(X_{i} \rightarrow Y) &=& \max\limits_{j}(precision(X_{i} \rightarrow Y_{j})\\ recall(X_{i} \rightarrow Y)&=&\max\limits_{j}(recall(X_{i} \rightarrow Y_{j}) \end{array} $$

Extending this notion to measure the similarity between all clusters from *X* to the clustering *Y*, we have:

$$\begin{array}{@{}rcl@{}} precision(X\rightarrow Y) &=& \frac {\sum\limits_{i} |X_{i}|precision(X_{i} \rightarrow Y)}{\sum\limits_{i}|X_{i}|}\\ recall(X\rightarrow Y) &=& \frac{\sum\limits_{i} |X_{i}|recall(X_{i} \rightarrow Y)}{\sum\limits_{i}|X_{i}|} \end{array} $$

where these values are weighted based on the sizes of the clusters inside *X* and *Y* such that the bigger clusters have a larger effect on the measures.

Now we can define the *F*1 score for similarity of *X* to *Y* by:

$$F1_{X \rightarrow Y} = \frac{2\times precision(X\rightarrow Y) \times recall(X\rightarrow Y)}{precision(X\rightarrow Y) + recall(X\rightarrow Y)}$$

Note that this measure only reflects a one-sided similarity. In other words, it finds the best matching cluster from *Y* to each cluster in *X* and gives an overall value of this. However, if for instance, *Y* is a superset of the input set, i.e., *Y* includes all possible clusters, then both *precision* and *recall* are going to be 100% while clearly *Y* is not a good clustering. To compensate for this problem we need to repeat the same operation for *Y*→*X* and average the results. Then for two clusterings *C*_1_ and *C*_2_, the average weighted *F*1 score is computed by:

$$F1 = \frac{F1_{C_{1}\rightarrow C_{2}} + F1_{C_{2}\rightarrow C_{1}}}{2}$$

The clustering generated so far is a non-overlapping clustering of the conserved regions. We can extend these clusters to their corresponding protein clusters by simply replacing each conserved region in a cluster by its originating protein. This can possibly result in some overlaps within different clusters.

### MapReduce implementation of graph construction

Construction of the similarity graph in each iteration of the Algorithm 1 can be performed using the MapReduce platform [8].

Because the graph generation algorit hm is called iteratively and in each call a set of *(k,c)-sketch*es are computed for the conserved regions using hash functions, in each iteration we can re-use the computed sketches from the previous iteration and aggregate them by the sketches computed using the required number of new hash functions. This can significantly improve the runtime of the process. In order to re-use the previously computed sketches, each conserved region needs to be assigned to a specific processor, and in each iteration the same assigned processor should be responsible for the new computation on that region.

A similar optimization can be performed for the computation of the second-level sketches. However, because adding new hash functions in the first-level can possibly add new neighbors to the input nodes for the second-level shingling, the computed sketches might need to be updated with regard to the new neighbors. This can be performed by storing the current neighbors list at each iteration so that the new neighbors can be identified and re-computation using previous hash functions can be avoided. This algorithm is demonstrated in Additional file 1: MapReduce algorithm for similarity graph construction.

Computation of the *F*1 score for clustering comparison can also be performed in parallel in the MapReduce framework. However, because this step is much faster than the clustering operation itself (and it is a rather intuitive algorithm), we forgo the details.

### Implementation and software availability

We have implemented our method using C++ together with the *MR-MPI* library [32] (version 7 April 2014) for MapReduce. Software is available as open source at:https://github.com/armenabnousi/minhash_clustering

## Results

### Experimental design

To evaluate our method we used a C++ implementation of our algorithm using the MR-MPI library [32]^3^ enabling MapReduce computation in an MPI environment. We ran our code on our in-house Aeolus 4 cluster with up to 128 (8 × 16) Intel processors (2.3 GHz, 126 Gb RAM on each node).

We used 9 different sets of proteins: 8 smaller sets of approximately 2000 protein sequences each (data sets #1-#8) and one larger set of approximately 90,000 protein sequences with 250,000 conserved regions annotated by Pfam (data set #9). Each of these sets contains various percentages of proteins from bacterial, archaeal, and eukaryotic domains. The composition and number of sequences in each of the smaller sets is presented in Table 1. For construction of each of these sets we randomly selected domain families from Pfam, extracted all the sequences that contained these domains (based on Pfam), agglomerated the sequences and removed redundant copies (if one sequence had multiple selected domains, only one copy of it was included in the final data set). Detailed lists of Pfam domain families constituting each of these sets, as well as the list of the Pfam domain families whose sets of sequences are used to construct data set #9, are presented in Additional file 2: Data Set Compositions. All operations in Pfam were performed using version 29 of the database.

**Table 1:** Composition of the smaller data sets (#1-#8)

Data set	# Sequences	% Bacteria	% Archaea	% Eukaryota
#1	1424	100%	0%	0%
#2	1542	100%	0%	0%
#3	1479	100%	0%	0%
#4	2037	95.4%	2.6%	2.0%
#5	808	93.1%	3.4%	3.5%
#6	2565	63.4%	1.2%	35.4%
#7	2138	29.5%	1.7%	68.8%
#8	1938	11.4%	1.8%	86.8%

We have assumed the domain families presented in Pfam (v.29) to be ground truth for clustering domain regions. However, as we will see, our method gives a higher resolution of clusters, more comparable to the results obtained from the pGraph/Grappolo pipeline introduced in [33], which we will henceforth refer to as pClust. In pClust [33], pGraph [34] is used for similarity graph generation using alignments, followed by Grappolo [31] for community detection on the generated graph.

For comparison between different clusters, we used the *F*1 score as defined earlier. This score is a modification of a measure used in another work on overlapping clustering [35]. The modification includes the addition of weights to give more importance to larger clusters and also the use of a two-sided computation with averaged results in contrast to the one-sided computation used in [35]. As described in the “Methods” section, the termination condition for the iterative process is based on the *F*1 score of the non-overlapping conserved region clusters. For all our data sets we used the Pfam domain regions as the input to our algorithm and to the pClust algorithm as well. Thus, a comparison between the non-overlapping clusters generated by these methods and by Pfam families was possible and because it was a lower level comparison, it was more accurate. On the other hand, to evaluate the overall performance of our NADDA - coreClust two-step pipeline approach for protein clustering, we performed another set of experiments where the inputs to our clustering method were the conserved regions found using NADDA. Because these regions do not necessarily match the Pfam domain regions, we were forced to perform the evaluation based on the extended, overlapping protein clusters rather than on the conserved region clusters.

For all computations of the *F*1 score (both during clustering iterations and evaluation) we ignored all clusters with fewer than 10 member sequences. In addition, for all clustering evaluation experiments we set the threshold for the Louvain method to 10^−7^.

Finally we performed a case-study by generating the phylogenetic network for 11 organisms using the data from [36] and approach presented in [33]. The motivation for this case-study was to show that our method would not only compare well with the computational results reported in [33], but importantly, would reflect the genetic relationships established by life scientists.

### Evaluation of the clusters

**Fig. 2:**
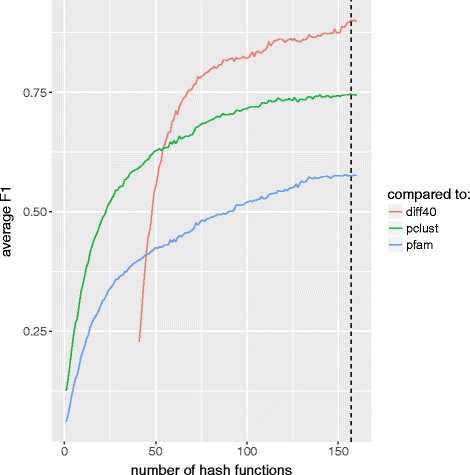
*F*1-value comparison for Pfam-annotated domains of data set #9 using different numbers of hash functions. For each iteration of the algorithm a comparison is made between Pfam and pClust (blue and green lines). The red line represents the *F*1-value computed at the end of each iteration using *d*=40. Comparisons are based on non-overlapping clusters of domain regions. The dashed line represents the number of hash functions where the termination condition is met for *τ*=0.9 and *d*=40

For each increment of the number of hash functions, our method generates a new set of clusters of the conserved regions until the termination condition is satisfied. Figure 2 shows the *F*1 score for the non-overlapping clusters of conserved regions computed in each increment of the hash function compared to the clusters generated using 40 fewer hash functions (the *F*1 score computed at the end of each iteration using *d*=40) for data set #9. The results are also compared to Pfam29 domain families and pClust clusters of the same domain regions. The figure demonstrates how incrementing the number of hash functions up to a certain point results in clusters that better resemble the output of Pfam/pClust. We use a threshold of *τ*=0.9 for the termination condition of our method, i.e., we stop incrementing the number of hash functions when comparison of the newly generated clusters to the ones generated by 40 fewer hash functions yields an *F*1 score of greater than 0.9. In Fig. 2, the termination condition is satisfied when using 157 hash functions.

Additionally, for each domain family present in more than 1000 sequences in data set #9 we have identified the best matching cluster from the coreClust results. For the 10 largest Pfam clusters, the fraction of the sequences from that domain family present in the matching clusters is shown in column 4 of Table 2. The complete set of results is presented in Additional file 3: Cluster Evaluation for Data Set #9. The ratio of the sequences in each of the matching clusters to the size of the cluster is shown in column 5 of this table. For each of these best matching clusters from coreClust we have also identified matching clusters using pClust results, and their corresponding fractions are shown in columns 7 and 8 of Table 2. It is noticeable that most of the fractions in column 5 are close to 1 (≥0.9). This implies that most of the clusters generated by coreClust tend to contain sequences that, based on Pfam, share a domain. However, the smaller fraction in column 4 of this table, implies that coreClust is breaking down the Pfam clusters into smaller subclusters. As we will see later (Fig. 5), coreClust captures a higher resolution subclusters from Pfam where each subcluster appears to correspond to a fraction of sequences that share a common domain and have a similar domain architecture. This is described in more detail later in this section. In this table multi-part domains in Pfam are counted multiple times because each part is input to coreClust as a separate conserved region.

**Table 2:** Comparison of the results for the 10 largest Pfam domain families in data set #9 with the output of coreClust and comparison of these coreClust clusters with their matching families based on pClust

Pfam family	|*Pfam*|	|*coreClust*|	$\frac {|Pfam \cap coreCl|}{|Pfam|}$	$\frac {|Pfam \cap coreCl|}{|coreCl|}$	|*pClust*|	$\frac {|pClust \cap coreCl|}{|pClust|}$	$\frac {|pClust \cap coreCl|}{|coreCl|}$
PF00397.23	9898	4504	0.45	0.999	5159	0.87	0.99
PF00109.23	9872	5185	0.52	0.99	11	1	0.002
					17	0.47	0.001
					108	0.75	0.01
					5149	0.92	0.91
PF02801.19	9861	5516	0.55	0.98	27	0.07	0.0003
					51	0.94	0.008
					39	0.02	0.0001
					10	0.3	0.0005
					5602	0.95	0.96
PF00400.29	7671	5566	0.71	0.98	36	0.78	0.005
					38	0.03	0.0001
					27	1	0.004
					11	1	0.001
					6781	0.80	0.97
PF13472.3	6708	957	0.12	0.84	10	1	0.01
					12	0.67	0.01
					460	0.64	0.31
					51	0.72	0.04
					441	0.85	0.39
					257	0.42	0.11
PF05729.9	6568	1351	0.20	0.997	43	0.98	0.03
					11	0.36	0.002
					24	0.75	0.01
					1322	0.96	0.94
PF16363.2	6325	3000	0.43	0.90	1725	0.98	0.56
					932	0.98	0.30
PF13516.3	6323	3206	0.50	0.996	3745	0.83	0.97
PF00550.22	6016	1486	0.23	0.94	14	0.5	0.004
					2125	0.61	0.88
					55	1	0.04
PF00053.21	5360	4422	0.75	0.91	4745	0.74	0.79
					112	0.98	0.02
					11	1	0.002
					41	0.76	0.01
					526	0.95	0.11

**Fig. 3:**
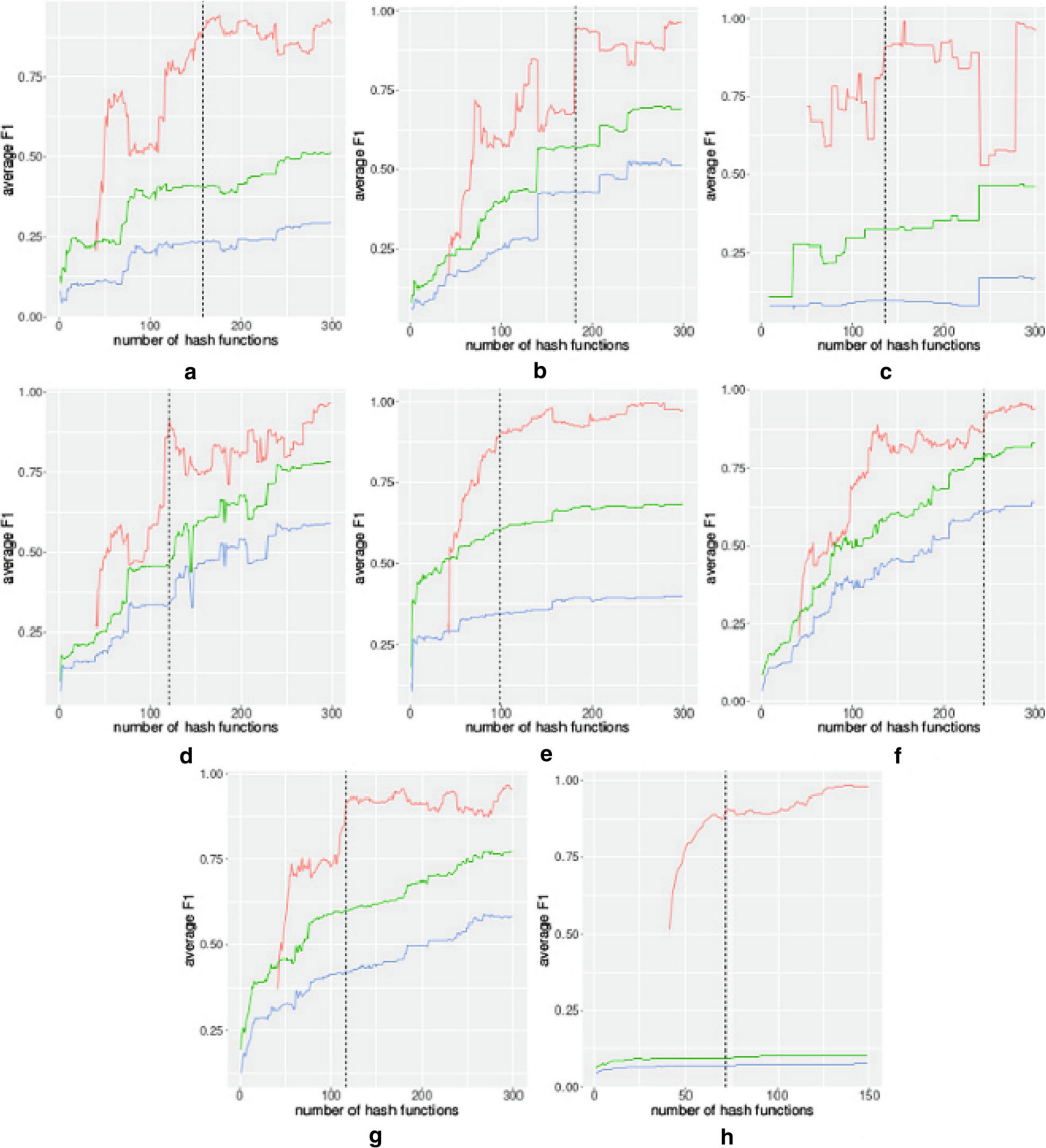
*F*1 score comparison for data sets #1-#8 using different numbers of hash functions. Red, green, and blue lines represent comparisons with the output of the algorithm for *h*−*d* hash functions, pClust, and Pfam clusters, respectively. Dashed lines in each plot show the number of hashes where the termination condition was satisfied (for *τ*=0.9 and *d*=40). **a** data set #1, **b** data set #2, **c** data set #3, **d** data set #4, **e** data set #5, **f** data set #6, **g** data set #7, **h** data set #8

**Fig. 4:**
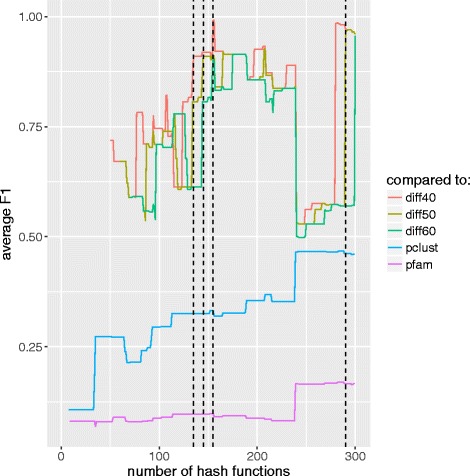
*F*1 score comparison for data set #3 using various values of *d* used in the termination condition. Red, olive, and green lines represent comparison at the end of each iteration with an earlier output of the algorithm using *d*=40, 50, and 60, respectively. Blue and purple lines demonstrate the comparison between pClust and Pfam clusters. Dashed lines represent the number of hash functions where the termination condition is satisfied, where from left to right the termination condition is (*τ*=0.9, *d*=40), (*τ*=0.9, *d*=50), (*τ*=0.9, *d*=60), and (*τ*=0.95, *d*=50)

Figure 3 shows a similar plot for each of the smaller sets (data sets #1-#8). Note that for smaller sets, a minor change in clustering due to the addition of a hash function has a more significant effect on the average *F*1 score and, hence, the more accented peaks and drops in these plots. These sudden increases and decreases in the *F*1 score can have an adverse effect in finding a proper number of hash functions where our method has converged, and increasing the number of hash functions does not benefit the output. To overcome this problem we can modify the parameters to the termination condition by either considering a larger threshold value for the termination condition (larger *τ*) or comparing the resulting clusters with a clustering obtained earlier than 40 hash functions before, for example, 50 hash functions (larger *d*). Using a larger threshold value will require us to stop later in the process when more hash functions are used. For example for data set #4, using *τ*=0.9 has resulted in stopping the process when reaching hash function 121 (the dashed line in Fig. 3d), while using a threshold of 0.95 would result in continuing to increment the number of hash functions up to 285. On the other hand, using a threshold of 0.95 would not result in a much different result for data set #3 due to the local maximum at hash number 156 (average *F*1=0.99). This can be accommodated by using a larger difference between the two compared clusters (larger *d*). Figure 4 shows the average *F*1 score when using *d*=40, 50, and 60 in the termination condition. Using a larger difference in the number of hash functions results in smaller *F*1 scores, avoiding premature termination of the process. In Fig. 4, using *τ*=0.9,*d*=50 causes the termination condition to hold when *h*=145 rather than 135 when we used *τ*=0.9,*d*=40. This number of hash functions increases to 155 when using *τ*=0.9,*d*=60 and to 290 when using *τ*=0.95,*d*=50.

**Fig. 5:**
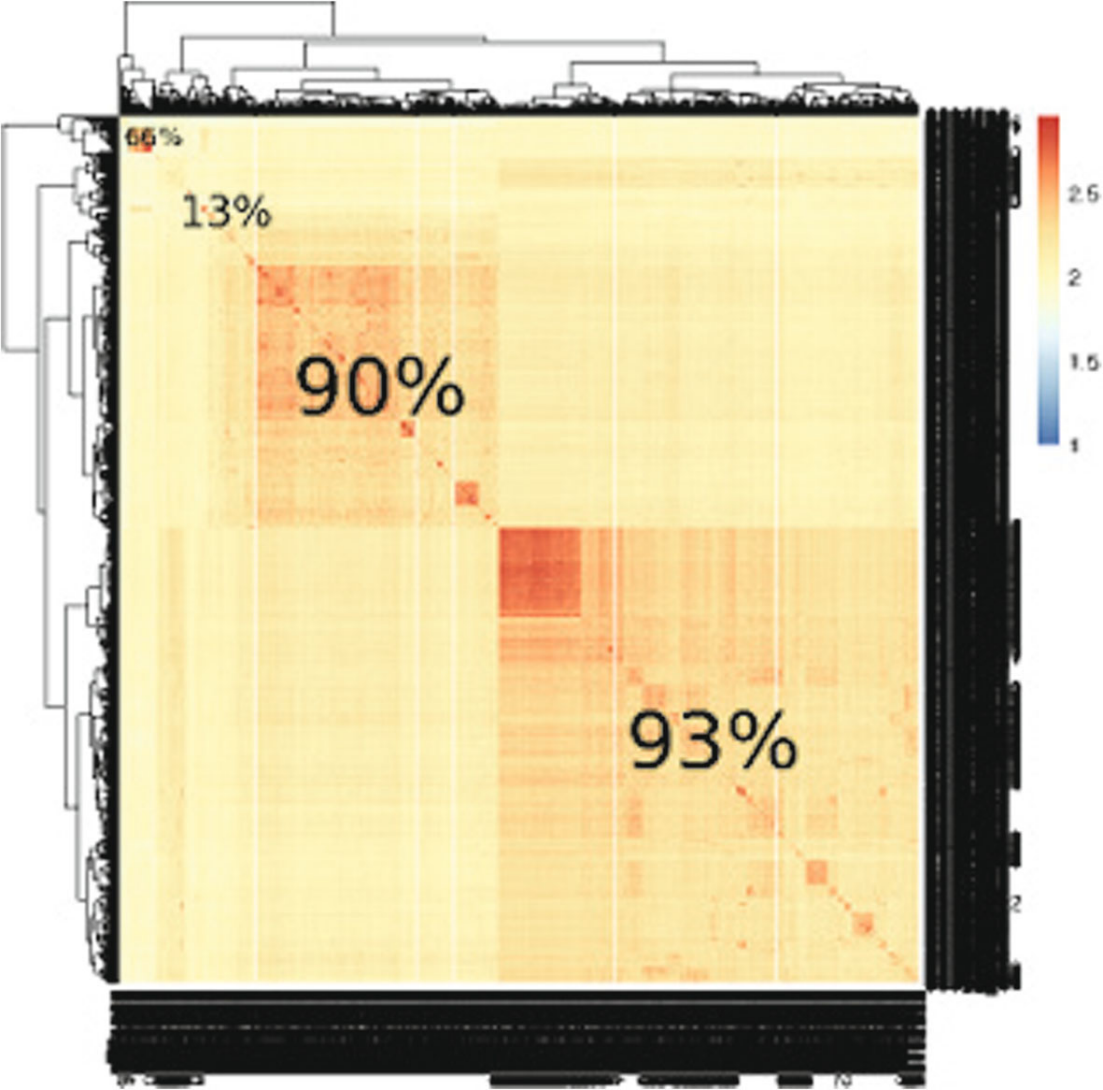
Heatmap generated based on the pairwise local similarity percentages of the sequences in the PF02801.19 domain family of Pfam. The darker rectangles represent sub-clusters that are more similar to each other than to the rest of the cluster. The overlaid percentages show the *F*1-value of the matching clusters from the output of our algorithm and the sub-clusters obtained by cutting the hierarchical clustering tree to generate four sub-clusters based on pairwise similarity scores. The *F*1 score of the matches from larger to smaller sub-clusters are 93%, 90%, 13%, and 66%

**Fig. 6:**
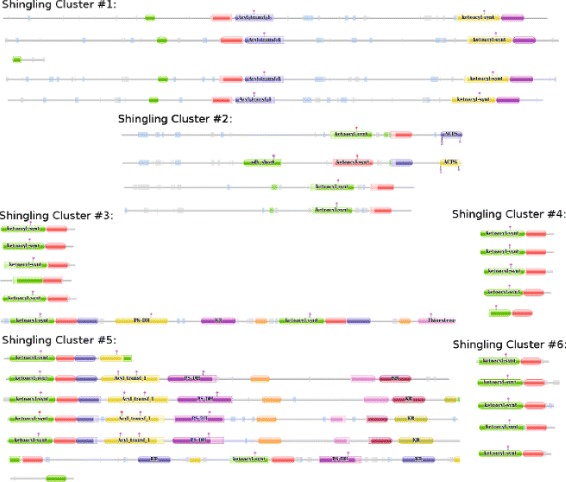
Shingling clusters matching with the PF02801.19 domain family of Pfam29. The sequences within each shingling cluster seem to have mostly the same architecture while differing from other clusters. This represents higher resolution clusters in our output compared to those of Pfam

We can also observe that our method results in a clustering more similar to the one obtained using pClust rather than Pfam29. As we briefly mentioned earlier, further investigation shows that our clustering gives a higher resolution of the clusters. In other words, some of the Pfam domain families can be broken down into smaller subfamilies where proteins within a subfamily are more similar to each other. We noticed that these subfamilies generally consist of proteins with a certain domain architecture, i.e., generally the collection of domains that are present in a protein are similar to each other within a subfamily but differ from the ones outside their subfamily. This is shown in Figs. 5 and 6 for the large data set (#9).

The heatmap in Fig. 5 shows the clustering generated based on pairwise scores obtained by applying Smith-Waterman sequence alignment on the entire set of sequences on one of the two largest Pfam domain families present in our data set (*PF*02801.19). To generate the clustering based on similarity scores we have used complete linkage hierarchical clustering (through the *pheatmap* package in *R*). The red rectangles represent groups of sequences that are more similar to each other than the remaining sequences in the same domain family. The numbers overlaid on these rectangles represent the *F*1 score of these sub-clusters when compared to the best matching cluster obtained coreClust. We can see that we have generated a clustering where these similar subclusters are captured rather than merging the entire domain family into one cluster.

In Fig. 6 we have randomly picked a few sequences from each of the clusters generated by our method that have matched with the *PF*02801.19 domain family, as described earlier, and then compared the architectures of these protein sequences with each other. We can see that the majority of the sequences in each cluster generated by our method follow a certain domain architecture.

Similar to Table 2, Table 3 demonstrates the fraction of matches for best matching clusters from coreClust to Pfam and from pClust to coreClust for data set #4. As is the case for data set #9, here also most of the coreClust clusters contain sequences that have a domain in common (column 5 of Table 3). Significantly, there is no outlier in these clusters as all fractions in column 5 equal 1. However, the fractions in column 4 tend to be smaller, implying that most of the sequences in their corresponding domain are not present in the identified best matching cluster. To demonstrate the recurrence of the phenomenon described earlier using the heatmap in Fig. 5, we have also provided Table 4. In this table we show all the coreClust clusters that match with 3 of the largest clusters from Pfam. As can be seen, most of the Pfam clusters are broken down into smaller coreClust clusters, where the coreClust clusters exclusively contain sequences from these clusters (fractions in column 5 equal 1 in all but one of the coreClust clusters). We can also see that for two of the three largest clusters of data set #4, most of the sequences from Pfam are contained in the matching set of clusters. Column 6 of Table 4 shows that 1084 of 1192 regions of *PF*00271.28 are contained in one of the clusters matching with this domain. Similarly, 1031 of the 1187 regions of *PF*00270.26 are contained in the clusters matching with this domain. However, only 341 regions from the 1232 regions of *PF*03880.12 (DbpA RNA binding domain) are clustered in matching clusters with this domain. Interestingly, the Pfam description of this domain states that “proteins can generally be distinguished by a basic region that extends beyond this domain [Karl Kossen, unpublished data]” [37, 38]. This may explain why this domain is not conserved enough to be captured and correctly clustered by our method.

**Table 3:** Comparison of the results for the Pfam domain families in data set #4 with the output of coreClust and comparison of these coreClust clusters with their matching families based on pClust

Pfam Family	|*Pfam*|	|*coreCl*|	$\frac {|Pfam \cap coreCl|}{|Pfam|}$	$\frac {|Pfam \cap coreCl|}{|coreCl|}$	|*pClust*|	$\frac {|pClust \cap coreCl|}{|pClust|}$	$\frac {|pClust \cap coreCl|}{|coreCl|}$
PF03880.12	1232	84	0.07	1	464	0.18	
PF00271.28	1192	364	0.30	1	1083	0.33	0.99
PF00270.26	1187	260	0.22	1	1077	0.24	1
PF08298.8	424	200	0.47	1	359	0.55	0.98
PF06798.9	410	172	0.42	1	199	0.86	1
PF04245.10	384	47	0.12	1	93	0.50	1
PF12343.5	24	15	0.62	1	24	0.62	1

**Table 4:** All clusters matching with the three largest Pfam clusters from data set #4. coreClust breaks down Pfam clusters into smaller clusters with zero to a few outliers

Pfam Family	|*Pfam*|	|*coreCl*|	$\frac {|Pfam \cap coreCl|}{|Pfam|}$	$\frac {|Pfam \cap coreCl|}{|coreCl|}$	Total
					clustered
					sequences
PF03880.12	1232	84	0.07	1	341
		54	0.04	1	
		44	0.03	1	
		27	0.02	1	
		24	0.02	1	
		17	0.01	1	
		15	0.01	1	
		15	0.01	1	
		14	0.01	1	
		13	0.01	1	
		13	0.01	1	
		11	0.01	1	
		10	0.01	1	
PF00271.28	1192	364	0.30	1	1084
		343	0.29	1	
		253	0.21	1	
		91	0.08	1	
		22	0.02	1	
		11	0.01	0.91	
PF00270.26	1187	260	0.22	1	1031
		245	0.21	1	
		196	0.16	1	
		141	0.12	1	
		105	0.09	1	
		72	0.06	1	
		12	0.01	1	

### Evaluation of the NADDA - coreClust

For evaluation of our clustering pipeline, we ran the NADDA domain detection algorithm on data set #9. Prior to running the NADDA domain detection algorithm on data set #9, however, it was necessary to train NADDA. To train the model used in NADDA we generated *k*-mer profiles for a protein data set of approximately 50,000 sequences, then we selected a representative set of around 3500 sequences from this set using CD-HIT40 [39], and finally we trained a random subspace model on the *k*-mer profiles of this subset as described in [4]. To train the model, a region in the training data is considered conserved if it exists in at least fifty sequences in the data set based on Pfam annotations. It should be noted that the resulting training set was completely distinct from data set #9.

After running data set #9 through NADDA, we eliminated regions with lengths shorter than 50 from the predicted conserved regions because these subsequences were less likely to represent a domain and more likely to be a result of random exact matches. The remaining conserved regions (about 205,000 regions) were fed to our coreClust algorithm for clustering.

**Fig. 7:**
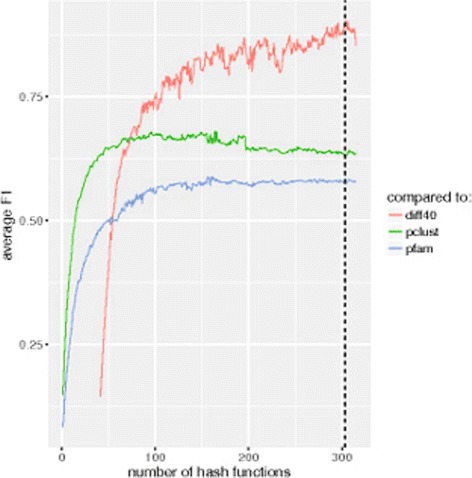
*F*1-value comparison for NADDA-annotated conserved regions of data set #9 using different numbers of hash functions. The red line represents the *F*1 score computed at the end of each iteration for checking with the termination condition (*d*=40). A comparison between Pfam and pClust protein clusters (overlapping) and the clustering of proteins generated at each iteration is shown with blue and red lines. The dashed line represents the number of hash functions where the termination condition is met (for *τ*=0.9 and *d*=40)

While in the previous evaluations we could use the non-overlapping clusters obtained from different algorithms, we cannot use the same approach here due to the differences in the input sets of conserved regions. Therefore, we extend the conserved regions’ clusters to protein sequences, disregarding the location of the conserved regions. The plot in Fig. 7 demonstrates the *F*1 score computed in different iterations of our process compared to the Pfam and pClust clusters. Using *τ*=0.9 and *d*=40 in the termination condition, we achieve an *F*1 score of 57% and 63%, respectively, compared to Pfam29 and pClust.

### Runtime evaluation

We have analyzed the runtime of our MapReduce implementation of the algorithm, and the results are presented in Additional file 4: Runtime Evaluation. A run with a higher resolution in hash functions completed processing the 90k input set (data set #9) in 90 min on 128 processors. We can achieve a near-linear speedup by employing a larger number of processors when increasing the number of hash functions in the iterations. It is important to note that the degree of conservation between proteins can affect the runtime by increasing the number of *k*-mer matches and therefore requiring more computation. In our experiments, the estimated conserved regions output by NADDA were clustered significantly faster than the domain regions annotated by Pfam. The runtime evaluation presented is based on Pfam regions.

### Case-study: phylogenetic network of Rickettsia

**Fig. 8:**
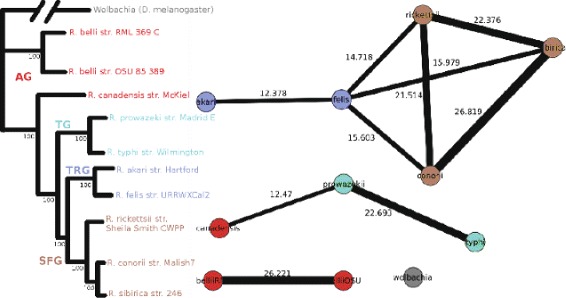
Phylogenetic network and tree of 10 Rickettisal organisms and an outlier. Demonstrated on the left is the tree generated by alignment in [36] and on the right is the network constructed based on our clustering method. Edge thickness in the network is proportional to the edge weight. Abbreviations: AG - ancestral group, TG - typhus group, TRG - transitional group, SFG - spotted fever group

In [36], the phylogenetic tree of 10 Rickettsial organisms and one outlier is constructed using alignment of 731 core representative proteins. In [33, 40] an approach for generation of the phylogenetic network of these organisms using clustering of their 13,571 proteins is presented. We have used their data set as input to the NADDA - coreClust two step pipeline to generate clusters of these proteins and then followed the method presented in [40] for construction of the adjacency matrix of the organisms and creation of a phylogenetic network. The result is presented in Fig. 8. The network is drawn using *visone* network visualization software [41] using the instructions presented in [40]. The thickness of the edges in the network presented in Fig. 8 is proportional to the edge weights. We can see that the tree structure is mostly maintained in the network. For example, the outlier organism (*Wolbachia*) is singled out from the rest of the nodes and the connectivity between all groups except for AG is conserved. Interestingly, for AG we see that *R. canadensis str. McKiel* is in a different subtree than the other two organisms of the same group, which is reflected in the network constructed based on our clustering. This seems to mirror the results obtained in [36] where there is some ambiguity between its relationship to the AG and TG branches.

## Discussion

The algorithm presented here for clustering protein sequences uses separate steps for conserved region detection and conserved region clustering. While this separation enables the evaluation and improvement of each step independently, we acknowledge that there are methods that use the output of the two steps iteratively to enhance the results. However, to the best of our knowledge, the present algorithm (accompanied with NADDA) is the first that permits complete separation of the tasks, and as such, it opens a new approach to studies in this field.

Our method depends heavily on the exact matching of *k*-mers. While we have shown that it can work well in most settings, there can be sets of proteins for which conservation is insufficient to allow enough exact matching *k*-mers of sufficient length. In our experiments we have used *k*=6, but there are some protein families where not enough exact matches of length 6 exist in their sequences. Using a smaller value for *k* also can be problematic since it may result in multiple random matches. However, we have shown in our experiments that for the most part, our proposed pipeline works well using *k*=6.

The more conserved a set of proteins is, the more exact matching *k*-mers exist in it. The increasing number of exact matches adds to the computation required by our algorithm, increasing its runtime. However, because our algorithm is parallelized in the MapReduce framework with a near-linear speedup, this shortcoming can be overlooked.

Finally, the selection of the big prime number *p* in the shingling step can be of importance. While most previous work mentions using a large *p*, no exact number is given. This is because the size of the selected prime number together with the range of the input values (*x* in *ax*+*b* mod *p*) can affect the outcome. A smaller *p* can result in more collisions in the hash table, which in turn may result in incorrect edges being inserted into the similarity graph. If the number of these incorrect edges is not significant, the clustering step (Grappolo) will be able to detect and reject them. On the other hand, a very large *p* can possibly lead to a collision-free hash table, which is most desirable, but given the size of the input dataset might be unachievable.

## Conclusions

Clustering of protein sequences is an important step in the prediction of protein function and structure. The proposed clustering method has multiple advantages. First it is only dependent on what can be thought of as the most basic information about proteins, i.e., their amino acid sequences. It also easily fits into the MapReduce framework permitting scalability and operation on large data sets. As discussed earlier, the separation of tasks proposed in this work and in our previous work [4] enables us and other researchers to focus on one problem at a time, analyze the results separately, and work towards optimizing and improving each one independently. Finally, our method does not require the alignment of sequences, a very time-consuming process, and as the availability of genomes continues to climb exponentially, this becomes increasingly more important for clustering methods.

In the future, it will be interesting to see the effect of using meta-sketches or super-shingles as proposed in [29], where second-level sketches are generated directly by hashing first-level sketches rather than by hashing the nodes of the graph generated in the first step. This may improve the runtime and memory usage at each step, but will likely require a larger number of hash functions and, thus, more iterations. Another possible direction for future work involves improving the termination condition, including automatic selection of the *τ* and *d* parameters. Finally, during the generation of the similarity graph in coreClust we draw an edge between two nodes if there is at least one hash function implying the existence of that edge (by giving equal minhashes for the two nodes). However, we can use the number of hash functions that imply the presence of an edge as a measure of weighting that edge. This information can be used to generate a weighted graph. Examining the performance of the algorithm using weighted graphs is an idea worth exploring.

## Additional files

Additional file 1 MapReduce algorithm for similarity graph construction (PDF 146 kb)

Additional file 2 Data Set Compositions (PDF 29 kb)

Additional file 3 Cluster Evaluation for data set #9 (PDF 74 kb)

Additional file 4 Runtime Evaluation (PDF 106 kb)

## Abbreviations

AG: Ancestral group
coreClust: Conserved region clustering
NADDA: Non-alignment domain detection algorithm
SFG: Spotted fever group
TG: Typhus group
TRG: Transitional group

## Footnotes

1 as of September 2017; release 2017_9

2 COnserved REgion CLUSTering

3
http://mapreduce.sandia.gov/index.html

4
https://docs.aeolus.wsu.edu/index.html
