# Supplementary material for: Alignment-free clustering of large data sets of unannotated protein conserved regions using minhashing
Source: BMC Bioinformatics. 2018 Mar 5;19:83. doi: 10.1186/s12859-018-2080-y (PMC5838936; doi:10.1186/s12859-018-2080-y)
Supplement: Supplementary file 1 — MapReduce algorithm for similarity graph construction (PDF 146 kb) [file 12859_2018_2080_MOESM1_ESM.pdf]

Additional File 1: MapReduce algorithm for similarity graph construction

In this algorithm, we have used the notation $< Key, Value >$ to denote the KeyValues in MapReduce and $< Key, [Value1, Value2, \ldots]$ to refer to the KeyMultiValues (shuffled KeyValues). For each Map and Reduce operation the input to the operation is shown inside parentheses in front of the name, and the output of the operation is separated by a colon (:).

Algorithm 1: Construction of the similarity graph in MapReduce

Input : S: set of conserved regions, h: current number of hash functions; $h-d$: previous number of hash functions
Output: G_h: A similarity graph for conserved regions

Function *CONSTRUCT_GRAPH_MAPREDUCE*($S, h, h-d$):
- `/* initialization: */`
- **if** *This is the first call to the procedure* **then**
  - Assign each conserved region to a processor;
  - Let S_{proc} denote the subset of S assigned to the processor $proc$;
  - **foreach** *conserved region* $S_i \in S_{proc}$ **do**
    - generate two empty tables t_{i1} and t_{i2} with zero columns and $c(=2)$ rows. Each row r is to represent, respectively, the first- and second-level sketches generated by the rth hash function.
  - **end**
- **end**
- `/* Main algorithm: */`
- `/* Generating initial graph using first-level sketches */`
- Send each conserved region to its assigned processor;
- Generate a new set of a, b, and p values;
- **foreach** $hn = (h-d+1)...h$ **do**
  - **foreach** $S_i \in S_{proc}$ *with id* $S_{i,id}$ **do**
    - Compute a new sketch using the new hash function hn, and store it in a new row in the table t_{i1};
  - **end**
- **end**
- **foreach** *sketch in* t_{i1} **do**
  - **Map** *(): emit* $< sketch, S_{i,id} >$
- **end**
- **Shuffle**;
- **Reduce** $(< sketch, [S_i, S_j, \ldots] >)$: $< S_i, S_j >, < S_j, S_i >, \ldots$;
- **Shuffle** `// sends KeyValue` $< S_i, S_j >$ `to the processor designated to process` S_i.
- `/* Generating second step graph based on common neighbors */`
- **foreach** *key* S_i *in the MapReduce* **do**
  - $Neighbors(S_i)$:= values of S_i;
  - $OldNeighbors(S_i)$:= retrieve the list of neighbors computed previously;
  - $NewNeighbors(S_i)$:= $Neighbors(S_i) - OldNeighbors(S_i)$;
  - **foreach** $hn = (h-d+1)...h$ **do**
    - Apply hash function hn on $NewNeighbors(S_i)$ and update the previous computed sketches (row hn on t_{i2});
  - **end**
  - **foreach** $hn = 1...h$ **do**
    - Apply hash function hn on $Neighbors(S_i)$ and compute new sketches;
  - **end**
  - **foreach** *sketch in* t_{i2} **do**
    - **Map** *(): emit* $< sketch, S_{i,id} >$;
  - **end**
  - **Shuffle**;
  - **Reduce** $(< sketch, [S_i, S_j, \ldots] >)$: $< S_i, S_j >, < S_j, S_i >$;
- **end**
- **foreach** *KeyValue* $< S_i, S_j >$ **do**
  - add an edge between the nodes S_i and S_j (if the edge does not exist);
- **end**
