# Supplementary material for: Alignment-free clustering of large data sets of unannotated protein conserved regions using minhashing
Source: BMC Bioinformatics. 2018 Mar 5;19:83. doi: 10.1186/s12859-018-2080-y (PMC5838936; doi:10.1186/s12859-018-2080-y)
Supplement: Supplementary file 2 — Data Set Compositions (PDF 29 kb) [file 12859_2018_2080_MOESM2_ESM.pdf]

Additional File 2: Dataset Compositions

Table 1 Selected families from Pfam to construct smaller data sets (#1-#8). Totals are computed after removing redundant copies of sequences.

(a) Data Set #1

Domain Name	# Sequences
FAD_binding_9	750
FixS	350
Gas_vesicle	368
total	1,424

(b) Data Set #2

Domain Name	# Sequences
Caa3_CtaG	499
Dak1_2	699
dCache_3	351
total	1,542

(c) Data Set #3

Domain Name	# Sequences
XisI	213
NapB	179
EutN_CcmL	330
LptC	823
total	1,479

(d) Data Set #4

Domain Name	# Sequences
NA37	384
DbpA	1,232
AAA_PrkA	426
total	2,037

(e) Data Set #5

Domain Name	# Sequences
NA37	384
AAA_PrkA	426
total	808

(f) Data Set #6

Domain Name	# Sequences
Rad4	693
YccF	1,034
DbpA	1,232
total	2,565

(g) Data Set #7

Domain Name	# Sequences
AAA_PrkA	426
DnaI_N	140
FTCD	262
FACT-Spt16_Nlob	433
SAD_SRA	890
total	2,138

(h) Data Set #8

Domain Name	# Sequences
Has-barrel	135
EccE	122
EFhand_Ca_insen	724
KA1	959
total	1,938

Table 2 Selected families from Pfam to construct large data set (#9). Total is computed after removing redundant copies of sequences.

Domain Name	# Sequences
PF00054.20	1,849
PF00081.19	2,349
PF00095.18	1,032
PF00397.23	6,345
PF00479.19	1,882
PF00961.16	265
PF01176.16	2,710
PF01220.16	1,314
PF01221.15	1,317
PF02198.13	1,046
PF02277.14	896
PF02735.13	1,289
PF02772.13	2,445
PF02801.19	8,583
PF03007.13	857
PF03106.12	3,025
PF03168.10	1,891
PF03306.10	318
PF03469.11	343
PF03629.15	779
PF03937.13	830
PF04383.10	1,803
PF04397.12	3,703
PF04863.10	66
PF04962.9	623
PF04963.10	954
PF05383.14	1,778
PF05729.9	6,534
PF06177.8	340
PF06414.9	524
PF06525.8	28
PF06824.8	501
PF07095.8	48
PF07179.9	500
PF07422.10	113
PF07677.11	1,068

Domain Name	# Sequences
PF07983.10	2,115
PF08059.10	728
PF08311.9	580
PF08314.8	305
PF08529.8	1,559
PF08712.8	760
PF08814.7	142
PF09043.8	91
PF09108.7	11
PF09383.7	1,134
PF10517.6	331
PF10537.6	439
PF10544.6	1,136
PF10599.6	52
PF11244.5	64
PF11764.5	362
PF12434.5	42
PF12971.4	325
PF13191.3	3,411
PF13472.3	6,696
PF13514.3	248
PF13629.3	403
PF13774.3	1,747
PF13808.3	313
PF13925.3	360
PF14524.3	425
PF14784.3	110
PF14804.3	561
PF14894.3	21
PF15901.2	814
PF16113.2	1,296
PF16363.2	6,294
PF16653.2	1,186
PF17047.2	628
total	94,599
