# Supplementary material for: Alignment-free clustering of large data sets of unannotated protein conserved regions using minhashing
Source: BMC Bioinformatics. 2018 Mar 5;19:83. doi: 10.1186/s12859-018-2080-y (PMC5838936; doi:10.1186/s12859-018-2080-y)
Supplement: Supplementary file 3 — Cluster Evaluation for data set #9 (PDF 74 kb) [file 12859_2018_2080_MOESM3_ESM.pdf]

Additional File 3: Cluster Evaluation for Data Set #9

Table 1: Comparison of the results for Pfam domain families with more than 1000 representations in data set #9 to the output of coreClust and comparison of these coreClust clusters to their matching families based on PClust.

Pfam Family	$\|Pfam\|$	$\|coreClust\|$	$\frac{\|Pfam \cap CoReCl\|}{\|Pfam\|}$	$\frac{\|Pfam \cap CoReCl\|}{\|CoReCl\|}$	$\|PClust\|$	$\frac{\|PClust \cap CoReCl\|}{\|PClust\|}$	$\frac{\|PClust \cap CoReCl\|}{\|CoReCl\|}$
PF00397.23	9898	4504	0.45	0.999	5159	0.87	0.99
PF00109.23	9872	5185	0.52	0.99	11	1	0.002
					17	0.47	0.001
					108	0.75	0.01
					5149	0.92	0.91
PF02801.19	9861	5516	0.55	0.98	27	0.07	0.0003
					51	0.94	0.008
					39	0.02	0.0001
					10	0.3	0.0005
					5602	0.95	0.96
PF00400.29	7671	5566	0.71	0.98	36	0.78	0.005
					38	0.03	0.0001
					27	1	0.004
					11	1	0.001
					6781	0.80	0.97
PF13472.3	6708	957	0.12	0.84	10	1	0.01
					12	0.67	0.01
					460	0.64	0.31
					51	0.72	0.04
					441	0.85	0.39
					257	0.42	0.11
PF05729.9	6568	1351	0.20	0.997	43	0.98	0.03
					11	0.36	0.002
					24	0.75	0.01
					1322	0.96	0.94
PF16363.2	6325	3000	0.43	0.90	1725	0.98	0.56
					932	0.98	0.30
PF13516.3	6323	3206	0.50	0.996	3745	0.83	0.97
PF00550.22	6016	1486	0.23	0.94	14	0.5	0.004
					2125	0.61	0.88
					55	1	0.04
PF00053.21	5360	4422	0.75	0.91	4745	0.74	0.79
					112	0.98	0.02
					11	1	0.002
					41	0.76	0.01
					526	0.95	0.11
PF16197.2	4661	3762	0.78	0.97	11	0.09	0.0002
					10	0.4	0.001
					32	0.12	0.001
					24	0.96	0.006
					10	1	0.002
					228	0.88	0.05
					14	0.07	0.0002
					2752	0.86	0.63
					11	0.91	0.002

					1050	0.86	0.24
PF00698.18	4510	3402	0.68	0.91	62	0.92	0.02
					29	0.72	0.006
					13	0.08	0.0002
					18	0.5	0.002
					1122	0.88	0.29
					299	0.62	0.05
					15	0.67	0.002
					19	0.05	0.0002
					24	0.62	0.004
					34	0.5	0.004
					131	0.43	0.01
					1967	0.82	0.48
					148	0.66	0.03
PF08659.7	3841	2606	0.62	0.92	13	0.61	0.003
					10	0.6	0.002
					32	1	0.01
					18	0.28	0.001
					23	0.65	0.005
					23	0.04	0.0003
					30	0.6	0.006
					10	0.2	0.0007
					61	0.87	0.02
					3248	0.70	0.88
PF04397.12	3705	463	0.12	0.99	12	0.08	0.002
					10	0.4	0.008
					587	0.36	0.46
					584	0.26	0.33
					12	0.33	0.008
PF03106.12	3527	3422	0.97	0.999	145	1	0.04
					3352	0.97	0.95
PF14765.3	3472	684	0.19	0.98	15	0.53	0.01
					28	0.71	0.03
					613	0.79	0.71
					177	0.56	0.14
					19	0.68	0.02
					10	0.3	0.004
					11	0.09	0.001
					10	0.7	0.01
					12	1	0.02
PF13191.3	3400	468	0.11	0.79	27	0.92	0.05
					235	0.25	0.13
					28	1	0.06
					35	0.71	0.05
					375	0.73	0.58
PF00054.20	3094	202	0.06	1	50	0.9	0.22
					208	0.71	0.73
PF00072.21	2966	1630	0.47	0.86	10	0.4	0.002
					22	0.5	0.006
					2151	0.65	0.86
					51	1	0.03
PF00008.24	2768	2141	0.66	0.85	31	0.77	0.01
					19	1	0.008

					30	0.87	0.01
					2727	0.67	0.85
					11	1	0.005
					11	0.90	0.004
PF01176.16	2710	1724	0.63	0.99	1739	0.98	0.99
					24	0.83	0.01
PF01846.16	2523	671	0.25	0.95	402	0.75	0.45
					40	0.75	0.04
					365	0.92	0.50
PF02772.13	2447	2405	0.97	0.99	2416	0.97	0.98
					13	1	0.005
PF00168.27	2441	933	0.36	0.94	824	0.93	0.82
					109	0.98	0.11
					38	1	0.04
PF02773.13	2416	2422	0.99	0.99	20	1	0.008
					2389	0.996	0.98
PF00438.17	2412	2353	0.97	0.999	2381	0.98	0.99
					18	1	0.007
PF00081.19	2351	2212	0.93	0.99	2292	0.94	0.98
PF02777.15	2324	2333	0.95	0.94	51	0.82	0.02
					2297	0.95	0.94
					42	1	0.02
					15	0.47	0.003
PF07983.10	2180	1949	0.88	0.98	18	0.94	0.008
					12	0.42	0.002
					1822	0.94	0.88
					20	0.5	0.005
					301	0.55	0.08
PF02210.21	2170	188	0.09	1	198	0.95	1
PF03168.10	1974	342	0.17	0.98	271	0.52	0.41
					233	0.80	0.54
PF00479.19	1891	1805	0.94	0.98	27	0.18	0.002
					1764	0.95	0.93
					69	0.97	0.04
PF02781.13	1887	1686	0.89	0.99	1762	0.91	0.95
					65	0.89	0.03
PF12796.4	1818	568	0.25	0.80	18	0.11	0.003
					878	0.50	0.77
					10	0.7	0.01
PF04383.10	1802	1013	0.56	1	1072	0.94	0.999
PF05383.14	1779	1596	0.86	0.96	1707	0.89	0.96
					56	0.71	0.02
PF08529.8	1662	1080	0.64	0.98	12	0.33	0.003
					109	0.80	0.08
					106	0.99	0.10
					1098	0.79	0.80
PF00095.18	1637	366	0.20	0.89	1289	0.25	0.88
					32	0.9375	0.08
PF00057.15	1608	1085	0.67	0.999	1567	0.69	0.999
PF13184.3	1555	1530	0.96	0.97	1553	0.96	0.97
					36	0.97	0.02
PF00957.18	1506	603	0.38	0.96	613	0.91	0.92
PF00595.21	1447	311	0.21	1	39	0.97	0.12

					286	0.95	0.88
PF00005.24	1367	957	0.67	0.96	10	0.1	0.001
					921	0.99	0.95
					39	1	0.04
PF01221.15	1350	843	0.62	0.99	976	0.85	0.98
PF16113.2	1340	1318	0.78	0.79	138	0.51	0.05
					44	0.82	0.03
					16	0.87	0.01
					1125	0.87	0.75
PF01220.16	1314	1469	0.96	0.86	24	0.46	0.00
					1309	0.97	0.86
PF02735.13	1294	168	0.13	0.99	79	0.80	0.37
					104	0.94	0.58
PF14484.3	1245	1199	0.84	0.88	1108	0.95	0.88
					19	0.10	0.001
PF00041.18	1205	68	0.06	1	69	0.97	0.98
PF16653.2	1190	905	0.39	0.51	10	0.1	0.001
					482	0.89	0.48
					499	0.88	0.49
					23	0.17	0.004
PF09383.7	1139	106	0.09	1	232	0.45	0.99
PF03435.15	1137	373	0.33	1	371	1	0.99
PF10544.6	1125	653	0.58	1	651	1	0.996
PF00632.22	1085	1081	0.99	0.99	1077	0.99	0.99
PF13855.3	1085	726	0.48	0.72	13	1	0.02
					27	0.63	0.02
					619	0.83	0.71
PF07677.11	1073	380	0.35	0.99	632	0.57	0.95
PF08242.9	1054	1050	0.78	0.79	30	0.07	0.001
					1038	0.80	0.79
					196	0.88	0.16
					28	0.11	0.002
PF07678.11	1049	751	0.56	0.78	32	1	0.04
					33	0.18	0.007
					604	0.81	0.65
					70	1	0.09
					18	1	0.02
PF02198.13	1046	168	0.16	0.99	176	0.94	0.98
PF00332.15	1029	491	0.47	0.98	562	0.78	0.89
					30	0.9	0.05
PF15901.2	1008	565	0.56	1	16	0.125	0.003
					149	0.86	0.23
					115	0.67	0.14
					203	0.98	0.35
					193	0.76	0.26
PF00069.22	1008	365	0.30	0.83	462	0.64	0.81
					59	1	0.16
