# Supplementary material for: Alignment-free clustering of large data sets of unannotated protein conserved regions using minhashing
Source: BMC Bioinformatics. 2018 Mar 5;19:83. doi: 10.1186/s12859-018-2080-y (PMC5838936; doi:10.1186/s12859-018-2080-y)

Additional File 4: Runtime Evaluation

We ran our algorithm on our in-house cluster (Aeolus[1]) using 32, 64, and 128 processors from 8 Intel nodes (2.3 GHz) with 126GB shared memory on each node. Using 128 processors and the input data set of domain regions extracted by Pfam from the 90,000 sequences (data set #9) took about 90 minutes for our algorithm to complete. However, this is when we used an increment of one in the number of hash functions used in each iteration. In practice, one does not need to use all possible hash functions, but can rather use, for example, increments of 20, which would decrease the runtime to 25 minutes. Furthermore, one might choose to start from a larger number of hash functions in the first iteration rather than using 41 hash functions.

The main contributor to runtime in our algorithm is the similarity graph generation step. The graph clustering step uses Grappolo[2] which is a multi-threaded implementation of the Louvain clustering method. We performed runtime analysis for the similarity graph generation using 32, 64, and 128 processors. Figure 1 shows the runtime required for each iteration of the algorithm for $d = 40$. At each iteration two similarity graphs should be generated, one for h hash functions and the other for $h - d$ hash functions. The drop in hash function 81 is because starting from $h = 81$, the similarity graph for $h - d$ hash functions has already been generated in earlier iterations and stored on disk. We can see that using 64 and 128 processors, significantly reduces the runtime compared to fewer number of processors. The variability in runtime for various numbers of hash functions can be partly due to the structure of the graph but is mainly a result of our cluster setup because various runs of the same algorithm also gave very variable runtimes.

The speedup for the similarity graph generation step is shown in Figure 2. To compute the speedup we have first computed the total runtime of all iterations for 32, 64, and 128 processors. Then we have divided the total runtimes by the total runtime using 32 processors. The speedup is sub-linear. However, a closer investigation shows that as we increase the number of iterations the speedup also improves. The dotted line in Figure 2 shows the speedup when the total runtime is computed only based on the iterations where $h > 80$. This is because scalability of our algorithm is based on the independence of hashing operations that can be spread out on different processors. If the number of hash functions used is not big enough, the communication time between processors will be the dominant contributor to runtime. One way to avoid this problem is to use a fewer number of processors in the first iterations of the algorithm and gradually increase this number as the algorithm proceeds to iterations with more hash functions. Please note that, as stated earlier, one can use increments of larger than one in between iterations, thus reducing the number of iterations with a smaller number of hash functions and contributing to the overall speedup. Note that at the time of the composition of this paper, Grappolo did not provide an API for calling the clustering functions from within our program. Therefore we store the generated similarity graph in each iteration of the algorithm on disk and then call Grappolo giving the disk on file as input. This can negatively affect the total runtime of the process.

[1] https://aeolus.wsu.edu
[2] https://github.com/luhowardmark/GrappoloTK

Figure 1 Runtime of similarity graph generation, broken down by iteration, using varying number of processors.

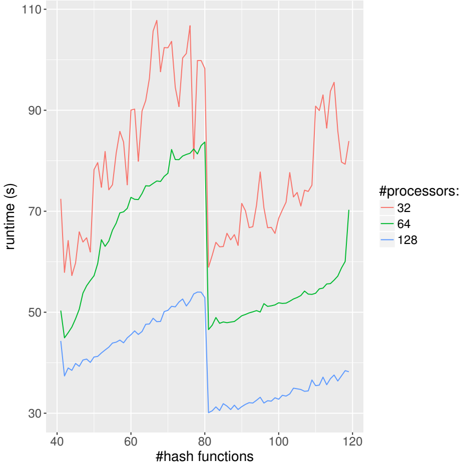

Figure 2 Speedup for similarity graph generation. The dashed line represents linear speedup that we would ideally expect. Solid line is the speedup resulted by considering all iterations of the algorithm for computing the total runtime, while the dotted line is the speedup when we have used only iterations with $h > 80$ in computation of the total time.

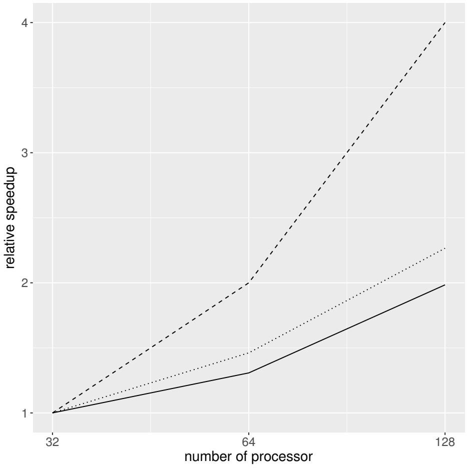

Supplement: Supplementary file 4 — Runtime Evaluation (PDF 106 kb) [file 12859_2018_2080_MOESM4_ESM.pdf]
